# Rietveld refinement of the langbeinite-type mixed-metal phosphate K_2_Ni_0.5_Zr_1.5_(PO_4_)_3_

**DOI:** 10.1107/S1600536814013658

**Published:** 2014-06-18

**Authors:** Igor V. Zatovsky

**Affiliations:** a Department of Inorganic Chemistry, Taras Shevchenko National University, 64/13, Volodymyrska St, 01601 Kyiv, Ukraine

## Abstract

Dipotassium [nickel(II) zirconium(IV)] tris­(orthophosphate) was prepared from a self-flux in the system K_2_O–P_2_O_5_–NiO–K_2_ZrF_6_. The title compound belongs to the langbeinite family and is built up from two [*M*O_6_] octa­hedra [*M* = Ni:Zr with mixed occupancy in ratios of 0.21 (4):0.79 (4) and 0.29 (4):0.71 (4), respectively] and [PO_4_] tetra­hedra inter­linked *via* vertices into a ^3^_∞_[*M*_2_(PO_4_)_3_] framework. Two independent K^+^ cations are located in large cavities of the framework, with coordination numbers to O^2−^ anions of nine and twelve. The K, Ni, and Zr sites are located on threefold rotation axes.

## Related literature   

For the structure of the mineral langbeinite, see: Zemann & Zemann (1957 ▶). For langbeinite-related phosphates based on different pairs of polyvalent metals, see: Wulff *et al.* (1992 ▶) for K_2_ *RE*Zr(PO_4_)_3_ (*RE* = Y, Gd); Orlova *et al.* (2003 ▶) for K_2_FeZr(PO_4_)_3_; Ogorodnyk *et al.* (2007*a* ▶) for K_1.96_Mn_0.57_Zr_1.43_(PO_4_)_3_ and K_1.93_Mn_0.53_Hf_1.47_(PO_4_)_3_; Ogorodnyk *et al.* (2007*b* ▶) for K_2_Ni_0.5_Ti_1.5_(PO_4_)_3_. For the profile function used in the Rietveld refinement, see: Thompson *et al.* (1987 ▶).

## Experimental   

### 

#### Crystal data  

K_2_Ni_0.5_Zr_1.5_(PO_4_)_3_
*M* *_r_* = 529.29
Cubic, 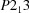

*a* = 10.15724 (13) Å
*V* = 1047.92 (2) Å^3^
*Z* = 4
Cu *K*α radiation, λ = 1.540598 Å
*T* = 293 K
Flat sheet, 25 × 25 mm

#### Data collection  

Shimadzu LabX XRD-6000 diffractometer
Specimen mounting: glass container
Data collection mode: reflection
Scan method: step
2θ_min_ = 10.910°, 2θ_max_ = 104.911°, 2θ_step_ = 0.020°

#### Refinement  

*R*_p_ = 0.100
*R*_wp_ = 0.134
*R*_exp_ = 0.034
*R*_Bragg_ = 0.041
*R*(*F*) = 0.035
χ^2^ = 15.761
4701 data points
107 parameters
2 restraints

### 

Data collection: *PCXRD* (Shimadzu, 2006 ▶); cell refinement: *DICVOL-2004* (Boultif & Louër, 2004 ▶); data reduction: *FULLPROF* (Rodriguez-Carvajal, 2006 ▶); program(s) used to solve structure: *FULLPROF*; program(s) used to refine structure: *FULLPROF*; molecular graphics: *DIAMOND* (Brandenburg, 1999 ▶); software used to prepare material for publication: *PLATON* (Spek, 2009 ▶) and *enCIFer* (Allen *et al.*, 2004 ▶).

## Supplementary Material

Crystal structure: contains datablock(s) global, I. DOI: 10.1107/S1600536814013658/wm5021sup1.cif

Rietveld powder data: contains datablock(s) I. DOI: 10.1107/S1600536814013658/wm5021Isup2.rtv

CCDC reference: 1007892

Additional supporting information:  crystallographic information; 3D view; checkCIF report

## Figures and Tables

**Table 1:** Selected bond lengths (Å)

K1—O1^i^	2.956 (16)
K1—O2^ii^	3.165 (14)
K1—O4^ii^	3.325 (14)
K2—O3^ii^	2.973 (15)
K2—O2^iii^	3.026 (16)
K2—O4^ii^	3.127 (15)
K2—O4^iii^	3.332 (15)
Zr1—O1	2.070 (14)
Zr1—O2^iv^	2.098 (14)
Zr2—O4	2.036 (12)
Zr2—O3^i^	2.041 (16)
P1—O3	1.530 (18)
P1—O4	1.523 (13)
P1—O2	1.515 (15)
P1—O1	1.493 (16)

Symmetry codes: (i) 
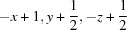
; (ii) 
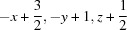
; (iii) 
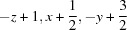
; (iv) 
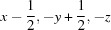
.

**Fig. 1.**
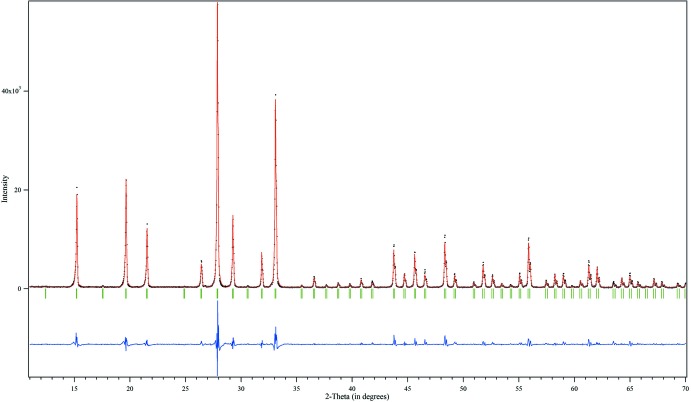
Results of the Rietveld refinement of K2Ni0.5Zr1.5(PO4)3. Experimental (dots), calculated (red curve) and difference (blue curve) data.

**Fig. 2.**
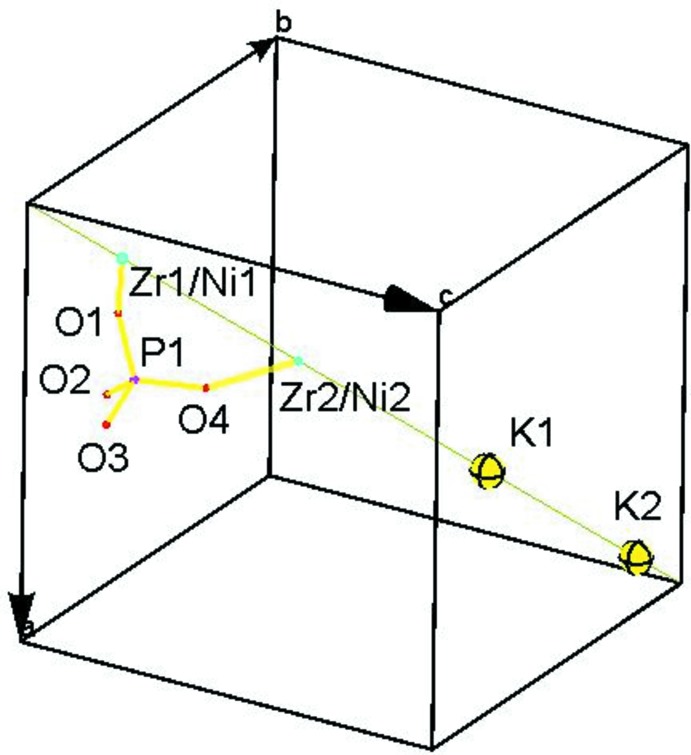
A view of the asymmetric unit of K2Ni0.5Zr1.5(PO4)3. Displacement ellipsoid are drawn at the 50% probability level.

**Fig. 3.**
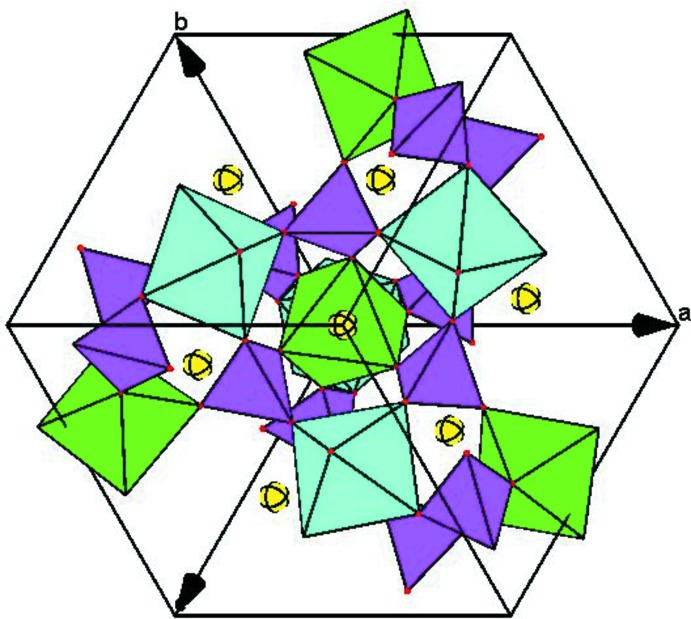
A projection of the structure of (I) along [111]. PO4 tetrahedra are pink, (Zr,Ni)1O6 octahedra are turquoise, (Zr,Ni)2O6 octahedra are green, K+ cations are shown as yellow spheres.

**Fig. 4.**
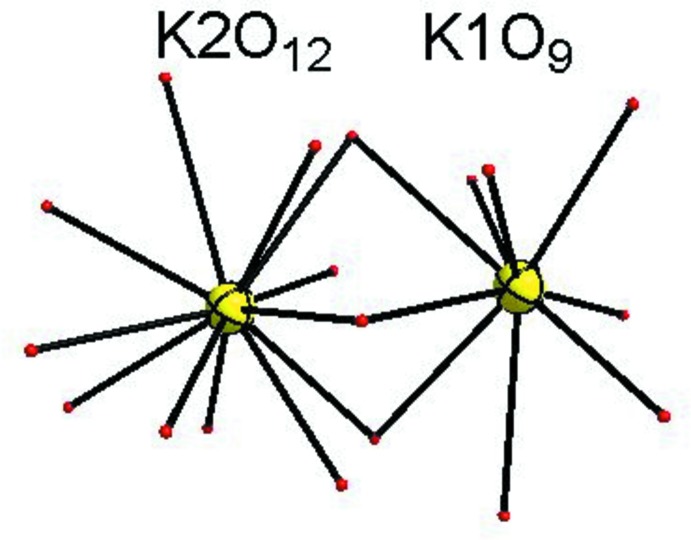
The O environment of K1+ and K2+ cations for (I). Displacement ellipsoid are drawn at the 50% probability level.

## supplementary crystallographic information

### S1. Comment

Phosphates of the langbeinite structure type are considered as favorable for environmentally safe crystalline forms of radioactive waste solidification (Orlova *et al.*, 2003). Langbeinite-type frameworks ^3^_∞_[*M*_2_(PO_4_)_3_] can be composed of various polyvalent metal pairs, for example, K_2_Ni_0.5_Ti_1.5_(PO_4_)_3_ (Ogorodnyk *et al.*, 2007*b*), K_1.96_Mn_0.57_Zr_1.43_(PO_4_)_3_ and K_1.93_Mn_0.53_Hf_1.47_(PO_4_)_3_ (Ogorodnyk *et al.*, 2007*b*), K_2_FeZr(PO_4_)_3_ (Orlova *et al.*, 2003), K_2_*RE*Zr(PO_4_)_3_, *RE* = Y, Gd (Wulff *et al.*, 1992). Herein the powder X-ray refinement of a phosphate, structurally isotypic with the mineral langbenite, K_2_Mg_2_(SO_4_)_3_ (Zemann & Zemann, 1957), K_2_Ni_0.5_Zr_1.5_(PO_4_)_3_, (I), is presented (Fig. 1).

The K, Ni, and Zr sites lie on threefold rotation axes in positions 4 *a* with the sequence {(Zr,Ni)1—(Zr,Ni)2—K1—K2} where (Zr,Ni)1 and (Zr,Ni)2 are metal sites with a mixed occupancy (Fig. 2). P and O atoms are located in 12 *b* positions.

The structure of (I) contains two independent [(Zr,Ni)O_6_] octahedra and one [PO_4_] tetrahedron which are linked together *via* common vertices, forming a three-dimensional framework (Fig. 3). The (Zr,Ni)–O bond lengths are 2.070 (14) Å, 2.098 (14) Å and 2.036 (12) Å, 2.041 (16) Å for [(Zr,Ni)1O_6_] and [(Zr,Ni)2O_6_], respectively. It should be noted that the occupancy of the metal sites by Ni^2+^ is slightly different (0.21 (4) for the *M*1 site and 0.29 (4) for the *M*2 site) whereas in case of K_2_Ni_0.5_Ti_1.5_(PO_4_)_3_ (Ogorodnyk *et al.*, 2007*b*) Ni^2+^ ions are almost equally distributed (occupancy of 0.25 for both positions), with ((Ti,Ni)—O bonds ranging from 1.938 (5) to 1.962 (5) Å. The three-dimensional framework ^3^_∞_[(Zr,Ni)_2_(PO_4_)_3_] has large closed cavities where the two independent K^+^ cations are located. K1 is coordinated by nine O atoms, while K2 is surrounded by twelve O atoms (Fig. 4), with K—O bond lengths ranging from 2.956 (16) to 3.332 (15) Å (Table 1).

### S2. Experimental

A well-ground mixture of 11.8 g KPO_3_ and 1.12 g NiO was placed in a platinum crucible and then was heated up to 1273 K. The temperature was kept constant during one hour and after that it was decreased to 1173 K. 4.25 g of K_2_ZrF_6_ were added to the flux under stirring with a platinum stirrer (initial K:P, Zr:P and Zr:Ni ratio equal to 1.3, 0.15, and 1.0, respectively). The crystallization of the melt was performed in the temperature range from 1173 to 913 K at an rate of 25 K/h. Finally, the crucible was cooled down to room temperature. The obtained material of (I) was recovered by washing with hot deionized water. The small crystals of (I) had the form of regular tetrahedra and were of light-yellow colour. The atomic ratio of the elements in (I) was found to be 4:1:3:6 for K/Ni/Zr/P, respectively: The sample was dissolved in 80% sulfuric acid under heating. The amount of the elements was then determined by atomic emission spectroscopy with inductive coupled plasma, AES-ICP, Spectroflame Modula ICP "Spectro".

### S3. Refinement

The powder pattern of (I) was indexed in the cubic system using *DICVOL-2004* (Boultif & Louër, 2004). The pattern indexing showed that the sample was a single phase. Atomic coordinates of K_1.96_Mn_0.57_Zr_1.43_(PO_4_)_3_ (Ogorodnyk *et al.*, 2007*a*) were used during Rietveld refinement as a starting model. For profile refinement a pseudo-Voigt function with axial divergence asymmetry (Thompson *et al.*, 1987) was used. First, the scaling factor, background, cell parameters *etc*. were refined during profile matching. Atomic coordinates were then refined during the next step. Atomic coordinates and displacement parameters of corresponding Zr and Ni sites were constrained to be the same. Isotropic displacement parameters of all atoms were appended to the refinement. The occupancies of K, Ni and Zr were refined taking into account that the occupancies of the hexacoordinated metal site should be equal to unity which was done using occupancy constraints. As the occupancy of the K sites was found to be 1, the occupancy factors of K1 and K2 were fixed at 1. The displacement factors of the O atoms were spread over a large range which is meaningless in this case due to the quality of the powder diffraction data. Thus *U*_iso_ values for all O atoms were constrained to be equal. As a result, the values of *U*_iso_ and their e.s.d.'s have close values. At the final refinement cycles two geometric restraints were applied to the lengths of P—O bonds because their values were unsatisfactory for the model (without restraints, one was ≈ 1.44 Å while another was close to 1.57 Å). Experimental, calculated and difference patterns are shown in Fig. 1.

### Crystal data

**Table:**

K_2_Ni_0.5_Zr_1.5_(PO_4_)_3_	*D*_x_ = 3.355 Mg m^−^^3^
*M**_r_* = 529.29	Cu *K*α radiation, λ = 1.540598 Å
Cubic, *P*2_1_3	*T* = 293 K
Hall symbol: P 2ac 2ab 3	Particle morphology: isometric
*a* = 10.15724 (13) Å	yellow
*V* = 1047.92 (2) Å^3^	flat sheet, 25 × 25 mm
*Z* = 4	Specimen preparation: Prepared at 293 K and 101.3 kPa

### Data collection

**Table:**

Shimadzu LabX XRD-6000 diffractometer	Data collection mode: reflection
Radiation source: X-ray tube, X-ray	Scan method: step
Graphite monochromator	2θ_min_ = 10.910°, 2θ_max_ = 104.911°, 2θ_step_ = 0.020°
Specimen mounting: glass container	

### Refinement

**Table:**

*R*_p_ = 0.100	107 parameters
*R*_wp_ = 0.134	2 restraints
*R*_exp_ = 0.034	9 constraints
*R*_Bragg_ = 0.041	Standard least squares refinement
*R*(*F*) = 0.035	(Δ/σ)_max_ = 0.001
χ^2^ = 15.761	Background function: Linear Interpolation between a set background points with refinable heights
4701 data points	Preferred orientation correction: March-Dollase Numeric Multiaxial Function
Profile function: Thompson-Cox-Hastings pseudo-Voigt * Axial divergence asymmetry	

### Special details

**Table:**

Geometry. Bond distances, angles *etc*. have been calculated using the rounded fractional coordinates. All su's are estimated from the variances of the (full) variance-covariance matrix. The cell e.s.d.'s are taken into account in the estimation of distances, angles and torsion angles

### Fractional atomic coordinates and isotropic or equivalent isotropic displacement parameters (Å^2^)

**Table:**

	*x*	*y*	*z*	*U*_iso_*/*U*_eq_	Occ. (<1)
K1	0.7043 (6)	0.7043 (6)	0.7043 (6)	0.054 (5)*	
K2	0.9317 (7)	0.9317 (7)	0.9317 (7)	0.052 (4)*	
Zr1	0.1448 (2)	0.1448 (2)	0.1448 (2)	0.007 (2)*	0.79 (4)
Zr2	0.4146 (3)	0.4146 (3)	0.4146 (3)	0.004 (2)*	0.71 (4)
Ni1	0.1448 (2)	0.1448 (2)	0.1448 (2)	0.007 (2)*	0.21 (4)
Ni2	0.4146 (3)	0.4146 (3)	0.4146 (3)	0.004 (2)*	0.29 (4)
P1	0.4581 (6)	0.2296 (8)	0.1286 (7)	0.004 (2)*	
O1	0.3180 (14)	0.2335 (14)	0.0844 (15)	0.003 (2)*	
O2	0.5417 (12)	0.2950 (14)	0.0238 (14)	0.003 (2)*	
O3	0.5025 (12)	0.0869 (16)	0.1471 (13)	0.003 (2)*	
O4	0.4729 (14)	0.3039 (12)	0.2580 (10)	0.003 (2)*	

### Atomic displacement parameters (Å^2^)

**Table:**

	*U*^11^	*U*^22^	*U*^33^	*U*^12^	*U*^13^	*U*^23^
?	?	?	?	?	?	?

### Geometric parameters (Å, º)

**Table:**

K1—O1^i^	2.956 (16)	Zr1—O1^xiii^	2.070 (14)
K1—O2^ii^	3.165 (14)	Zr1—O2^xiv^	2.098 (14)
K1—O4^ii^	3.325 (14)	Zr2—O4	2.036 (12)
K1—O1^iii^	2.956 (16)	Zr2—O3^i^	2.041 (16)
K1—O2^iv^	3.165 (14)	Zr2—O4^xi^	2.036 (12)
K1—O4^iv^	3.325 (14)	Zr2—O3^iii^	2.041 (16)
K1—O1^v^	2.956 (16)	Zr2—O4^xiii^	2.036 (12)
K1—O2^vi^	3.165 (14)	Zr2—O3^v^	2.041 (16)
K1—O4^vi^	3.325 (14)	Ni1—O2^xii^	2.098 (14)
K2—O3^ii^	2.973 (15)	Ni1—O1^xiii^	2.070 (14)
K2—O2^vii^	3.026 (16)	Ni1—O2^xiv^	2.098 (14)
K2—O4^ii^	3.127 (15)	Ni1—O1^xi^	2.070 (14)
K2—O4^vii^	3.332 (15)	Ni1—O1	2.070 (14)
K2—O3^iv^	2.973 (15)	Ni1—O2^x^	2.098 (14)
K2—O2^viii^	3.026 (16)	Ni2—O4	2.036 (12)
K2—O4^iv^	3.127 (15)	Ni2—O3^v^	2.041 (16)
K2—O4^viii^	3.332 (15)	Ni2—O3^i^	2.041 (16)
K2—O3^vi^	2.973 (15)	Ni2—O4^xi^	2.036 (12)
K2—O2^ix^	3.026 (16)	Ni2—O3^iii^	2.041 (16)
K2—O4^vi^	3.127 (15)	Ni2—O4^xiii^	2.036 (12)
K2—O4^ix^	3.332 (15)	P1—O3	1.530 (18)
Zr1—O1	2.070 (14)	P1—O4	1.523 (13)
Zr1—O2^x^	2.098 (14)	P1—O2	1.515 (15)
Zr1—O1^xi^	2.070 (14)	P1—O1	1.493 (16)
Zr1—O2^xii^	2.098 (14)		
			
O1—Zr1—O2^x^	93.2 (5)	O1—Ni1—O2^x^	93.2 (5)
O1—Zr1—O1^xi^	90.6 (6)	O1—Ni1—O1^xi^	90.6 (6)
O1—Zr1—O2^xii^	175.9 (5)	O1—Ni1—O2^xii^	175.9 (5)
O1—Zr1—O1^xiii^	90.6 (6)	O1—Ni1—O1^xiii^	90.6 (6)
O1—Zr1—O2^xiv^	87.7 (6)	O1—Ni1—O2^xiv^	87.7 (6)
O1^xi^—Zr1—O2^x^	87.7 (6)	O1^xi^—Ni1—O2^x^	87.7 (6)
O2^x^—Zr1—O2^xii^	88.6 (5)	O2^x^—Ni1—O2^xii^	88.6 (5)
O1^xiii^—Zr1—O2^x^	175.9 (5)	O1^xiii^—Ni1—O2^x^	175.9 (5)
O2^x^—Zr1—O2^xiv^	88.6 (5)	O2^x^—Ni1—O2^xiv^	88.6 (5)
O1^xi^—Zr1—O2^xii^	93.2 (5)	O4^xi^—Ni2—O4^xiii^	87.5 (5)
O1^xi^—Zr1—O1^xiii^	90.6 (6)	O3^v^—Ni2—O4^xi^	170.8 (5)
O1^xi^—Zr1—O2^xiv^	175.9 (5)	O3^iii^—Ni2—O4^xiii^	84.5 (5)
O1^xiii^—Zr1—O2^xii^	87.7 (6)	O3^iii^—Ni2—O3^v^	92.1 (5)
O2^xii^—Zr1—O2^xiv^	88.6 (5)	O3^v^—Ni2—O4^xiii^	96.5 (5)
O1^xiii^—Zr1—O2^xiv^	93.2 (5)	O3^i^—Ni2—O3^iii^	92.1 (5)
O3^i^—Zr2—O4	96.5 (5)	O3^i^—Ni2—O4	96.5 (5)
O4—Zr2—O4^xi^	87.5 (5)	O4—Ni2—O4^xi^	87.5 (5)
O3^iii^—Zr2—O4	170.8 (5)	O3^iii^—Ni2—O4	170.8 (5)
O4—Zr2—O4^xiii^	87.5 (5)	O4—Ni2—O4^xiii^	87.5 (5)
O3^v^—Zr2—O4	84.5 (5)	O3^v^—Ni2—O4	84.5 (5)
O3^i^—Zr2—O4^xi^	84.5 (5)	O3^i^—Ni2—O4^xi^	84.5 (5)
O3^i^—Zr2—O3^iii^	92.1 (5)	O3^iii^—Ni2—O4^xi^	96.5 (5)
O3^i^—Zr2—O4^xiii^	170.8 (5)	O3^i^—Ni2—O4^xiii^	170.8 (5)
O3^i^—Zr2—O3^v^	92.1 (5)	O3^i^—Ni2—O3^v^	92.1 (5)
O3^iii^—Zr2—O4^xi^	96.5 (5)	O3—P1—O4	109.5 (8)
O4^xi^—Zr2—O4^xiii^	87.5 (5)	O1—P1—O2	108.1 (9)
O3^v^—Zr2—O4^xi^	170.8 (5)	O1—P1—O3	110.1 (9)
O3^iii^—Zr2—O4^xiii^	84.5 (5)	O1—P1—O4	109.9 (9)
O3^iii^—Zr2—O3^v^	92.1 (5)	O2—P1—O3	109.7 (8)
O3^v^—Zr2—O4^xiii^	96.5 (5)	O2—P1—O4	109.5 (9)
O1^xi^—Ni1—O2^xii^	93.2 (5)	Zr1—O1—P1	135.2 (10)
O1^xi^—Ni1—O1^xiii^	90.6 (6)	Ni1—O1—P1	135.2 (10)
O1^xi^—Ni1—O2^xiv^	175.9 (5)	Zr1^xv^—O2—P1	168.8 (10)
O1^xiii^—Ni1—O2^xii^	87.7 (6)	Zr2^xvi^—O3—P1	153.7 (9)
O2^xii^—Ni1—O2^xiv^	88.6 (5)	Zr2—O4—P1	156.8 (9)
O1^xiii^—Ni1—O2^xiv^	93.2 (5)	Ni2—O4—P1	156.8 (9)

Symmetry codes: (i) −*x*+1, *y*+1/2, −*z*+1/2; (ii) −*x*+3/2, −*y*+1, *z*+1/2; (iii) −*z*+1/2, −*x*+1, *y*+1/2; (iv) −*y*+1, *z*+1/2, −*x*+3/2; (v) *y*+1/2, −*z*+1/2, −*x*+1; (vi) *z*+1/2, −*x*+3/2, −*y*+1; (vii) −*z*+1, *x*+1/2, −*y*+3/2; (viii) −*y*+3/2, −*z*+1, *x*+1/2; (ix) *x*+1/2, −*y*+3/2, −*z*+1; (x) *x*−1/2, −*y*+1/2, −*z*; (xi) *z*, *x*, *y*; (xii) −*z*, *x*−1/2, −*y*+1/2; (xiii) *y*, *z*, *x*; (xiv) −*y*+1/2, −*z*, *x*−1/2; (xv) *x*+1/2, −*y*+1/2, −*z*; (xvi) −*x*+1, *y*−1/2, −*z*+1/2.
